# Factors Affecting Visual Gain after Accelerated Crosslinking in Pediatric Keratoconic Cases

**DOI:** 10.14744/bej.2021.15046

**Published:** 2021-12-17

**Authors:** Mehmet Tayfur, Serap Yurttaser Ocak, Mustafa Nuri Elcioglu

**Affiliations:** 1. Department of Ophthalmology, Akcakale State Hospital, Sanliurfa, Turkey; 2. Department of Ophthalmology, Okmeydani Prof. Dr. Cemil Tascioglu City Hospital, Istanbul, Turkey

**Keywords:** Accelerated collagen crosslinking, pediatric keratoconus

## Abstract

**Objectives:**

This study was designed to evaluate factors affecting visual gain following accelerated crosslinking (A-CXL) in pediatric cases with keratoconus.

**Methods:**

Pediatric patients with a diagnosis of keratoconus who underwent A-CXL for 10 minutes ultraviolet A (UV-A) 9 mW/cm^2^ between February 2015 and May 2019 and had at least 1 year of follow-up data were analyzed retrospectively. Uncorrected visual acuity, best corrected visual acuity (BCVA), and refractive value changes (spherical, cylindrical values) at the preoperative and postoperative 12^th^ month were compared. Correlation analysis was used to evaluate the relationships between visual gain and corneal topography values (K1, K2, Kmaximum [Kmax], anterior elevation, posterior elevation) obtained using a Sirius topography device (Costruzione Strumenti Oftalmici, Florence, Italy).

**Results:**

A total of 33 eyes of 22 patients (mean age: 16.85±1.15 years) who underwent A-CXL (9 mW/cm^2^ UVA irradiation for 10 minutes) were included in the study. The mean BCVA had increased from 0.45±0.27 logMAR to a mean of 0.33±0.24 logMAR at the end of 1 year (p=0.014). Changes to spherical and cylindrical values were not statistically significant (p>0.05). The correlation analysis revealed a statistically significant relationship between visual gain and the preoperative BCVA and the preoperative Kmax.

**Conclusion:**

The study results indicated that the visual gain at 1 year after A-CXL (10 minutes at 9 mW/cm^2^) was associated with preoperative BCVA and Kmax values in pediatric cases of keratoconus.

## Introduction

Keratoconus is characterized by progressive corneal thinning and steepening which can show bilateral but asymmetrical course between the eyes (1-3). Keratoconus typically presents in teenage years and progresses over the years (4). The disease progresses more rapidly during teenage years when compared with adults (5, 6). Therefore, to avoid corneal transplantation, it is essential to stop the progression of the keratoconus in childhood.

The conventional corneal collagen cross-linking method (CXL) has been demonstrated to stop the progression of the disease (2). In this method, the cornea is saturated with riboflavin drops for 30 min, and then 3 mW/cm^2^ UV-A is applied for 30 min. Cross-links occur between riboflavin and UV-A and corneal collagens (2). These bonds cause an increase in corneal rigidity, which is thought to play a role in stopping progression (2). Depending on the Bunsen-Roscoe law, which is used to explain photochemical reactions such as CXL treatment, the UV-A energy absorbed by the cornea shows its effectiveness in the tissue according to the total dose amount. The intensity of the applied energy and the application time can be changed so that the total amount of applied energy does not change. Therefore, the equal dose physical principle, 9 mW/cm^2^ in 10 min, 18 mW/cm^2^ in 5 min, 30 mW/cm^2^ in 3 min, and 45 mW/cm^2^ in 2 min, corresponding to 5.4 J/cm^2^ energy doses, is predicted to be as effective as the conventional CXL treatment with the Dresden protocol (7). Based on this law, accelerated methods have come to increase patient comfort, since the total duration of operation in conventional CXL is 1 h. Thus, A-CXL methods have been shown to have similar efficacy and reliability with conventional methods (3). However, the number of studies reporting results of accelerated cross-linking (9 mW/cm^2^ in 10 min) in pediatric patients in the literature is quite limited (8-10). These studies have demonstrated the safety and efficacy of accelerated CXL therapy in arresting the progression of keratoconus in pediatric patients. In these studies, a similar significant improvement in visual acuity after A-CXL was shown in pediatric keratoconus cases. To the best of our knowledge, there is no study on the factors affecting vision gain.

The aim in this study is to evaluate the factors affecting visual improvement in pediatric cases with keratoconus after A-CXL application (9 mW/cm^2^–10 min) at the end of 1-year follow-up.

## Methods

For the study, pediatric cases with progressive keratoconus disease who underwent A-CXL (9 mW/cm^2^–10 min) between February 2015 and May 2019 were retrospectively analyzed. Cases that had a follow-up period of at least 1 year were included in the study. The study was approved by the ethical committee of Okmeydani Prof. Dr. Cemil Tascioglu City Hospital according to the principles of the Declaration of Helsinki. The written informed consent was obtained from each patient before the procedure.

Patients over 18 years old, patients with a corneal thickness of <400 microns (μm), with scars or opacities in the cornea, with a history of ocular surgery or systemic disease, with ocular pathology except keratoconus, and cases with a <12 months follow-up were excluded from the study. Uncorrected visual acuity (UCVA), best-corrected visual acuity (BCVA), and change in refractive values (spherical and cylindrical) at pre-operative visit and post-operative 12^th^ months examination were statistically compared. The gain of BCVA was obtained by subtracting the pre-operative BCVA value (Snellen) from the BCVA value (Snellen) at post-operative 12^th^ months. The correlation analysis was used to evaluate the relationship between the visual improvement and the mean value of pre-operative parameters (UCVA, BCVA, and tomography parameters [K1: Flat keratometer, K2: Steep keratometer, Kmax: Maximum keratometer, Kvb: Keratoconus vertex back, Kvf: Keratoconus vertex front, TCT: Thinnest corneal thickness, and CCT: Central corneal thickness]). The tomography parameters were obtained from corneal tomography device – Sirius^®^ (Scheimpflug tomography system – CSO Inc.). The regression model was determined with BCVA Gain = 1.016–0.024 (Kmax) + 1.094 (BCVA-logMAR) formula; the BCVA values increase as the pre-treatment BCVA values decrease, and the Kmax values increase as the pre-treatment BCVA values decrease.

### Surgical Technique

A-CXL treatment protocol was administered under topical anesthesia (0.5% proparacaine hydrochloride (Alcaine^®^, Alcon Laboratories, Inc., Hünenberg, Switzerland) under sterile conditions. After debridement of the epithelial layer with a diameter of 8–9 mm in the central cornea, riboflavin drops (MedioCROSS^®^ M Kiel, Germany) containing 0.1% riboflavin and 1.1% hydroxypropyl methyl cellulose were dripped every 2 min for a duration of 20 min. Afterward, a UVA beam with a diameter of 9 mm (370 nm) was applied from a distance of 5 cm perpendicular to the corneal apex with the help of a device (Peschke Vario 365, Hunenberg, Switzerland). UVA duration was 10 min (9 mW/cm^2^, total 5.4 J/cm^2^). After the eye was washed with BSS (20 ml balanced salt solution) solution, a bandage contact lens was placed.

### Statistical Analysis

Number Cruncher Statistical System 2007 (Kaysville, Utah, USA) program was used for statistical analysis. While evaluating the study data, in addition to descriptive statistical methods (mean, standard deviation, median, frequency, ratio, minimum, and maximum), the repeated measure test was used for the repeated measurements of the variables with normal distribution, and the Friedman test was used for the repeated measurements of the variables that did not show normal distribution. The Wilcoxon test was used for the measurements of the variables that showed normal distribution. Pearson’s correlation analysis and Spearman correlation analysis were used to evaluating the relationships between quantitative variables. Significance was assessed at p<0.05 levels.

## Results

The study included 33 eyes of 22 cases; 63.6% (n=14) male and 36.4% (n=8) female. The mean age was 16.85±1.15 years (range 13–18).

Table 1 shows the UCVA and BCVA values before the surgery and 12^th^ months after the surgery. The increase of UCVA and BCVA at post-operative 12^th^ months was statistically significant compared with the pre-treatment (p<0.001) situation.

**Table 1.** The Evaluation of Visual Acuity Values

n=33	Min-Max (Median)	Mean±SD
UCVA (logmar)		
Pre-CXL	0.1–1.5 (0.7)	0.68±0.36
Post CXL 12^th^ months	0–1.5 (0.5)	0.52±0.35
p		^a^0.010**
BCVA (logmar)		
Pre-CXL	0–1 (0.4)	0.45±0.27
Post CXL 12^th^ months	0–1 (0.3)	0.33±0.24
p		^a^0.011^**^

^a^Wilcoxon test. UCVA: Uncorrected visual acuity; BCVA: Best corrected visual acuity; CXL: Crosslinking; SD: Standard deviation.

There was no statistically significant difference between pre-operative and post-operative changes in refractive errors. Table 2 shows the changes in refractive errors preoperatively and at 12^th^ months postoperatively.

**Table 2.** The evaluation of refractive data at 12^th^ months after the surgery compared to the situation before the surgery

	Min/Max	Median	p
Spherical refractive value			
Pre-CXL	-11.75-1 (-1.75)	-2.73±3.17	
Post-CXL 12^th^ m	-9-2 (-2)	-2.71±2.79	^a^0.799
Cylindrical refractive value			
Pre-CXL	-1.01±1.66	1.57±12.51	
Post-CXL 12^th^ m	-0.8±1.61	-0.31±1.87	^a^0.124

aFriedman Test. CXL: Crosslinking.

Table 3 shows the correlation between the gain in BCVA and the mean values of pre-operative parameters (UCVA, BCVA, refractive errors, and topographic parameters [K1, K2, Kmax, Kvf, Kvb, CCT, and TCT]).

**Table 3.** The corrrelation of the preoperative UCVA, BCVA, refractive errors and topographic parameters with the gain in BCVA at the end of 1 year post-CXL

	Gain of BCVA
Pre-CXl	Mean±SD	^c^r	p
UCVA (logmar)	0.68±0.36	0.135	0.455
BCVA (logmar)	0.45±0.27	0.663	0.001^**^
Refractive errors			
SRV	-2.73±3.17	-0.444	0.510
CRV	1.57±12.51	-0.068	0.706
Topographic parameters			
Kmax	56.27±4.65	-0.244	0.040^*^
KVb	67.06±25.70	0.229	0.200
KVf	33.06±13.15	0.236	0.185
K1	45.24±2.43	0.280	0.114
K2	48.99±3.39	0.306	0.083
TCT	439.42±40.75	-0.100	0.579
CCT	453.79±40.25	-0.137	0.447

^c^r= Spearman Correlation Coefficient. ^*^P<0.05, ^**^p<0.01. K1: Flat keratometer; K2: Steep keratometer; Kmax: Maximum keratometer; Kvb: Keratoconus vertex back; Kvf: Keratoconus vertex front; TCT: Thinnest cornal thickness; CCT: Central corneal thickness; UCVA: Uncorrected visual acuity; BCVA: Best corrected visual acuity; SRV: Spherical refractive value; CRV: Cylindrical refractive value; CXL: Crosslinking; SD: Standard deviation.

A statistically significant correlation of 0.663 (r=0.663; p=0.001; p<0.01) was detected between the pre-treatment BCVA logMAR values and the amount of gain in BCVA, with a positive correlation relationship (the pre-treatment BCVA logMAR values increase as the gain in BCVA increases).

A negative statistically significant correlation (r=−0.244; p=0.040 p<0.05) was found between the pre-treatment Kmax values and the amount of gain in BCVA (as the Kmax values increase, the gain in BCVA decreases).

Table 4 shows the regression analysis of gain in BCVA with the mean values of pre-operative BCVA and Kmax.

**Table 4.** The regression analysis of pre-CXL BCVA and Kmax values with the gain in BCVA values

Pre-CXL	Unstandardized Coefficients		95.0% Confidence Interval for B	
	B	p	Lower Bound	Upper Bound
Kmax	-0.024	0.019^*^	-0.044	-0.004
BCVA	1.094	0.001^**^	0.752	1.437
(Constant)	1.016	0.056	-0.029	2.060

## Discussion

Keratoconus tends to progress rapidly, especially in pediatric cases. Thus, it is important to stop the progression of keratoconus in childhood.

The efficacy and safety of standard CXL therapy in pediatric cases have been demonstrated in many studies in the literature (11). Although optimal irradiation and timing have not been determined yet, A-CXL has become preferred in clinical practice because it shortens the duration of the treatment. This advantage is particularly important in the pediatric patient group, where collaboration and compliance are the biggest challenges during local surgical procedures. In addition to the short duration of treatment, the results of A-CXL in pediatric cases show that it is effective and safe (8-10). When both standard and accelerated CXL results were evaluated, it was determined that crosslinking had a positive effect on vision as well as stopping the progression of keratoconus (11). Our aim in this study was to evaluate the factors affecting the visual gain after A-CXL application (9 mW/cm^2^ – 10 min) at the end of 1 year follow-up in pediatric cases with keratoconus.

Ulusoy et al. (9) investigated the effects of A-CXL in 28 eyes (10 min of UVA irradiation with a total energy dose of 5.4 J/cm^2^ at 9 mW/cm^2^) in pediatric keratoconus cases at postoperatively 12^th^ months and showed an improvement in the mean BCVA over the follow-up period. Similar to this study, Badawi showed statistically significant improvement in BCVA 1 year after A-CXL in pediatric keratoconus cases (10). Shetty et al. (8) also reported 2-year follow-up results of A-CXL (10 min UVA irradiation at 9mW/cm^2^, total energy dose 5.4 J/cm^2^) in 30 eyes of 18 keratoconus patients under 14 years of age. They showed significant improvement in BCVA during the follow-up period. In our study, we found a statistically significant improvement in the mean BCVA value at 12^th^ months after the A-CXL compared with the pre-operative values (p=0.014).

To find the predictors in terms of BCVA gain; we applied correlation and regression analysis between the pre-operative variables and the BCVA gain at postoperatively 12^th^ months. According to these results, we found two predictors: pre-operative Kmax and pre-operative BCVA values.

An inverse correlation was found between the pre-operative Kmax values and the BCVA gain at post-operative 12^th^ months. Due to the rapid and severe progression of keratoconus in children and adolescents, most authors recommend not wasting time for observing the progression of keratoconus and applying the CXL treatment as soon as the definitive diagnosis is made (12). Our results also support the literature in this sense. As the pre-operative Kmax increases, the gain in BCVA decreases which makes us think that it would be a more rational approach to perform CXL as soon as the diagnosis is made. Therefore, applying CXL prevents cases from progressing to advanced stages.

The second predictor was the pre-operative BCVA values. A direct correlation was found between the pre-operative BCVA values and the gain in BCVA at postoperatively 12^th^ months. The BCVA gain increases as the pre-operative BCVA (logMAR) increases. These results show that CXL provides a positive benefit of the BCVA gain in cases with low vision.

The limitations of this study were its retrospective design, small number of cases, and limited follow-up time of 1-year.

## Conclusion

The gain in BCVA values at 12^th^ months after A-CXL was found to be associated with the pre-operative BCVA and maximum keratometry values in pediatric keratoconus cases.

### Disclosures

**Ethics Committee Approval:** Okmeydani Prof. Dr. Cemil Tascioglu City Hospital Ethics Committee, protocol number: 48670771-514.10, Date: 14/07/2020.

**Peer-review:** Externally peer-reviewed.

**Conflict of Interest:** None declared.

**Authorship Contributions:** Involved in design and conduct of the study (SYO, MNE, MT); preparation and review of the study (V SYO, MNE, MT); data collection (MT); and statistical analysis (SYO, MT).
